# On the TPPP Protein of the Enigmatic Fungus, *Olpidium*—Correlation between the Incidence of p25alpha Domain and That of the Eukaryotic Flagellum

**DOI:** 10.3390/ijms232213927

**Published:** 2022-11-11

**Authors:** Ferenc Orosz

**Affiliations:** Institute of Enzymology, Research Centre for Natural Sciences, 1117 Budapest, Hungary; orosz.ferenc@ttk.hu

**Keywords:** Blastocladiomycota, Chytridiomycota, flagellum, Olpidiomycota, p25alpha domain

## Abstract

Loss of the flagellum was an important step in the evolution of fungi. The flagellated fungi of the phylum Olpidiomycota are the closest relative of the non-flagellated terrestrial fungi. There are genes encoding proteins, the occurrence of which shows a strong correlation with the incidence of the flagellum. One of these gene/protein families is “TPPP-like proteins” whose main feature is the presence of the p25alpha domain. The functional link between TPPP and flagellum has also been shown. Most of the phyla of flagellated fungi have been known to contain TPPP-like proteins but Olpidiomycota was an exception. This study demonstrates that *Olpidium bornovanus*, similarly to some fungi of Chytridiomycota and Blastocladiomycota, has a “fungal-type” TPPP characterized by the presence of two (a complete and an incomplete) p25alpha domains.

## 1. Introduction

The early-branching fungi (i.e., which are near to the root of the tree) reproduce through the production of motile zoospores, propelled by a single, posteriorly oriented flagellum and are dependent on aquatic environment for dispersal [1]. However, more evolved terrestrial fungi “lost the flagellated zoospore stage and invented means to disperse the spores aerially” [2]. Loss of the flagellum was an important step in the evolution of fungi and was interpreted to be associated with the terrestrial radiation of non-flagellated fungi [3]. The loss of the flagellum might have either occurred once [4] or at least four times [5]. However, recent data by Chang et al. [2] support the first scenario. According to the recent classification by Tedersoo et al. [6], which adopts 18 phyla in Fungi, flagellated fungi can be found in seven phyla: Rozellomycota (alias Cryptomicota), Aphelidiomycota, Neocallimastigomycota, Monoblepharomycota, Chytridiomycota, Blastocladiomycota and Olpidiomycota.

Comparative genomic studies [7,8] revealed the existence of the so-called flagellar or ‘ciliary’ genes and proteins, which are present in all eukaryotic organism possessing flagella. Some of these genes/proteins belong to the family of TPPP-like proteins containing at least one p25alpha domain [9,10], which consists of about 160 amino acids [11,12]. (TPPP refers to the term “tubulin polymerization promoting protein” [13].) The p25alpha domain (Pfam05517, IPR008907) generally does not occur in non-flagellated species. The functional connection between TPPP and flagellum was proven in *Chlamydomonas reinhardtii*, a biflagellated green alga [14]. Its TPPP ortholog, FAP265 protein, can be found in the flagella, and is essential in its formation, as shown by using FAP265 null mutants [14]. TPPP-like proteins can be grouped according to two characteristics: (i) the length of their p25alpha domain, which can be long, short, truncated or partial; and (ii) the presence or absence of other type of domains [10]. (For example, apicortin contains both partial p25alpha and DCX domains [15].) Recently, a TPPP-form present only in Fungi has also been identified [16]. (See later).

A novel study has revealed that as expected, most of the flagellated fungi contain TPPP-like proteins [16]. Phyla Rozellomycota (Cryptomycota), Neocallimastigomycota, Monoblepharomycota, Chytridiomycota and Blastocladiomycota possess these proteins; however, they were not found in Aphelidiomycota and Olpidiomycota, probably due to the lack of enough genomic and proteomic data, either in general (NCBI) or in special (Mycocosm) databases. The Olpidiomycota contains only a single genus *Olpidium* which was placed among Zygomycota, species of which are non-flagellated [1,17]. It was suggested [16] that the apparent hiatus of TPPP-like proteins in *Olpidium* is due to its incomplete sequencing caused by technical problems [18]. However, a recent analysis has shown that in contrast to the above mentioned two studies where *Olpidium* was placed within non-flagellated terrestrial fungi, *Olpidium* is their sister group [2]. This topology substantiates a single loss of the flagellum rather than its multiple losses among the fungi [2]. The repositioning was possible since the authors of that study successfully sequenced the *Olpidium bornovanus* which provided sufficient data for the analysis. In this paper, I show that *O. bornovanus* has at least one TPPP-like (p25alpha domain containing) protein by analyzing sequence data recently made available on the NCBI website.

## 2. Results

**Figure 1:**
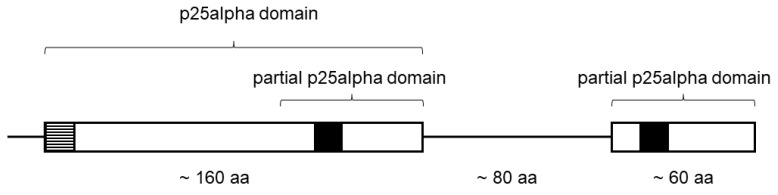
Schematic structure of a fungal-type TPPP. The positions of the Rossmann-like motifs (GXGXGXXGR) are indicated by black squares. The dashed line square indicates the LxxxF(Y)xxFxxF sequence. aa- amino acids.

TPPP-like proteins are characterized by the presence of the p25alpha domain [10]. It starts generally with a LxxxF(Y)xxFxxF sequence. The C-terminal part of the domain contains a very characteristic “Rossman-like” sequence, GxGxGxxGR (Figure 1) [10]. These proteins can be grouped on the basis of the length and completeness of the p25alpha domain and the presence of another kind of domain(s) [10]. A special, “fungal-type” TPPP, which contains both a full and a partial (C-terminal) p25alpha domain, is present only in certain Fungi (Figure 1) [16].

Blast analyses in NCBI databases (https://www.ncbi.nlm.nih.gov/protein/ and https://www.ncbi.nlm.nih.gov/nuccore, accessed on 4 November 2021) revealed the presence of p25alpha domain containing proteins and nucleotides in *O. bornovanus*. Two partial hypothetical proteins, KAG5460860 and KAG5458366, and two WGS (whole genome shotgun) sequences, JAEFCI010004592 and JAEFCI010008581, were found. KAG5460860 and KAG5458366 includes 64 and 163 amino acids, respectively. The amino acid sequences of KAG5460860 and KAG5458366 correspond partly to the JAEFCI010004592 and JAEFCI010008581 WGS sequences, respectively. JAEFCI010008581 encodes the C-terminal part of the KAG5458366 protein since the nucleotide bases coding the last amino acid of the protein are followed by a stop codon. The N-terminal half is missing and cannot be completed based on this nucleotide sequence. It seems that the beginning of this partial protein corresponds to a real exon boundary since the TPX65513 protein of *Chytriomyces confervae* (a fungal type TPPP) has an exon boundary exactly at this position.

**Figure 2:**
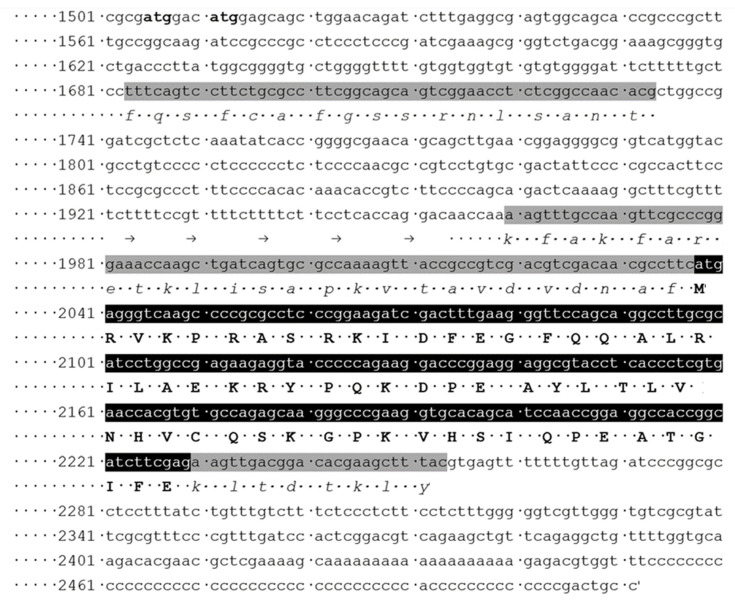
Partial sequence of *Olpidium bornovanus* isolate S191 BJ554k121_659286, whole genome shotgun sequence (GenBank: JAEFCI010004592.1). Numbers indicate the order of its nucleotides. Black background indicates the nucleotides corresponding to the KAG5460860, hypothetical protein. Bold capital letters show the amino acid sequence of this protein. Gray background indicates nucleotides whose translation are shown in Figure 3. The corresponding amino acids are shown with lower case italic letters. The possible positions of the initiation codon (atg) are labeled by bold letters.

**Figure 3:**
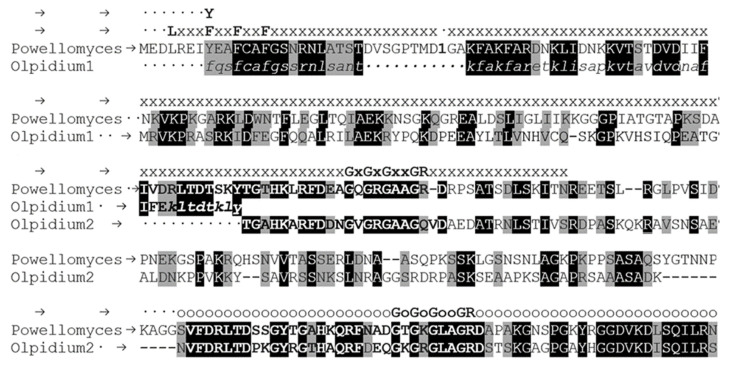
Alignment of *Olpidium bornovanus* and *Powellomyces hirtus* proteins by Clustal Omega [19]. *Olpidium*1—*O. bornovanus* KAG5460860, hypothetical protein, partial. *Olpidium*2—*O. bornovanus* KAG5458366, hypothetical protein, partial. Powellomyces—*P. hirtus* TPX57673, hypothetical protein. Lower case italic letters stand for amino acids of *Olpidium*1 obtained by manual translation of JAEFCI010004592 whole genome shotgun sequence. Bold letters stand for sequences being present in duplicates in the proteins. Identical and biochemically similar amino acids in both proteins are labeled by black and grey background, respectively. Letters ‘x’ and ‘o’ indicate the p25alpha and partial p25alpha domains, respectively. The “Rossman-like” sequences, GXGXGXXGR, and the LXXF(Y)XXFXXF sequence at the beginning of the p25alpha domain are also shown. The position of the first intron (phase 1) of the *P. hirtus* protein is marked by number 1. (The glycine is coded by the last nucleotide of the first exon and the first two nucleotide of the second one).

**Figure 4:**
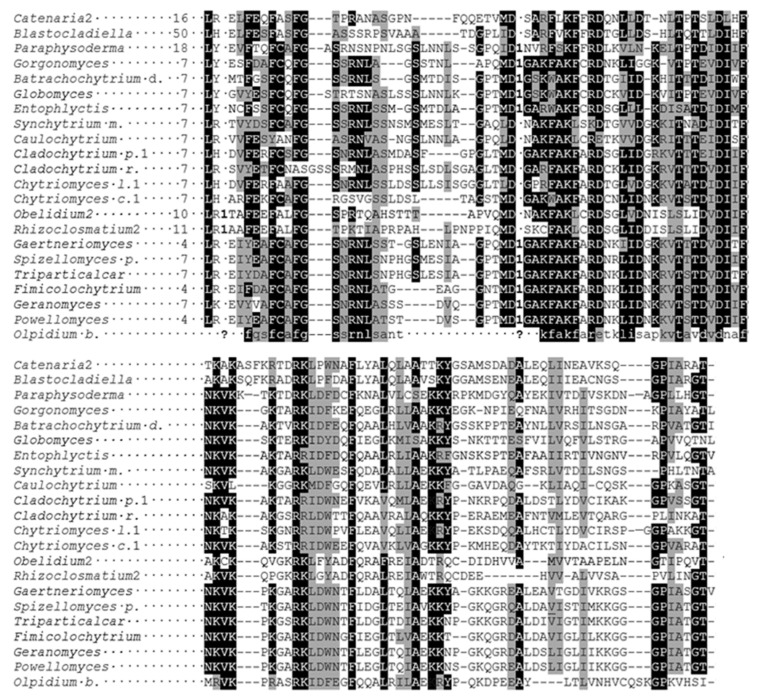

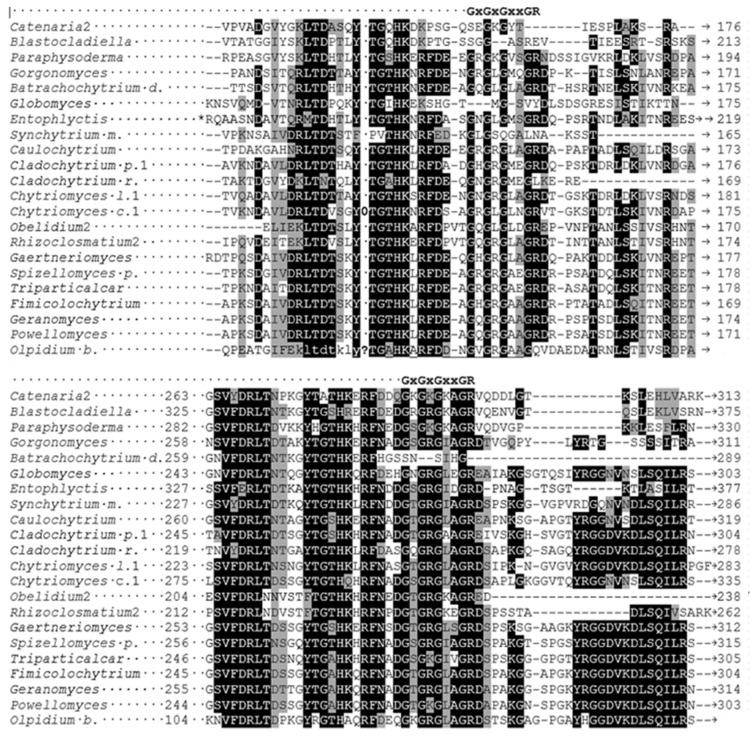
Multiple alignment (manually refined) of the p25alpha domains of fungal-type TPPPs by Clustal Omega [20]. The N-termini (amino acids before the p25alpha domain) and the interdomain parts are not included in the alignment. Amino acids that are identical and biochemically similar in at least two thirds of the proteins are labeled by black and grey background, respectively. The “Rossman-like” sequences, GXGXGXXGR, are also shown. The positions of a phase 1 intron of some proteins and that of a phase 0 intron of *Chytriomyces confervae* TPX65513 protein are marked by number 1 and 0, respectively. Asterisk (*) indicates that 39 amino acids of Enthel1467718 protein of *Entophlyctis helioformis* following the arginin (R) are not shown for clarity. Question marks (?) indicate the positions of the tentative introns of *Olpidium bornovanus* TPPP. The Accession Numbers of fungal proteins are listed in Table 1.

However, the translation of the JAEFCI010004592 sequence indicated that the partial sequence of the KAG5460860 hypothetical protein can be completed, at least partly. At the C-terminal end of the partial protein it can be done with certainty (nucleotides 2230-2253) (Figure 2), but not at the N-terminus. KAG5460860 starts with a methionine coded by nucleotides 2038-2040 of JAEFCI010004592. The previous nucleotides were translated manually (Figure 2). Two parts of the translated sequence resulted in amino acid sequences which are highly homologous to known fungal-type proteins (cf. Figure 3). One of such sequences can be found immediately before the starting methionine and coded by nucleotides 1960–2037; the other one is coded by nucleotides 1683–1733 (Figure 2). There is no homology in the middle region (nucleotides 1734–1959). It should be noted that the numbers of nucleotides (226) in this intermediate region cannot be divided by three, there is a phase shift here between the two coding regions, so it is very likely that these nucleotides represent an intron. It is common in most fungal-type TPPPs that the first ~30–40 amino acids are encoded by an exon separated by a phase 1 intron from the second exon (in all members of the classes of Spizellomycetes and Rhizophydiomycetes, and also in *Paraphysoderma sedebokerense*), which can also be the case here (Figure 4). However, the very N-terminal part of the first exon is missing; the homologous translated sequence starts with the fifth amino acid of the p25alpha domain, and no initiation codon can be identified. The N-terminus may be encoded by another exon, but we cannot say for sure.

The possible starting methionine may be encoded by triplets 1505–1507 or 1511–1513 (Figure 2). There are examples of an intron with such a position; in *Rhizoclosmatium globosum* ORY45507 and *Obelidium mucronatum* Obemuc1859513 fungal-type TPPPs; there are phase 1 introns here in both cases (Figure 4). Thus *O. bornovanus* TPPP is encoded probably by four exons. Based on the combination of the partly supplemented sequence of KAG5460860 and the sequence of KAG5458366, a more complete but still an incomplete hypothetical protein sequence can be produced, the very N-terminal part of which is still absent (Figure 3). Using this sequence as query in BLASTP search of the NCBI website, fungal-type TPPPs of various fungi were obtained as best hits (Table 2). Each of them belongs to the Chytridiomycota phylum. TPX57673 protein of *P. hirtus* was found as the most similar one. The alignment of the *Olpidium* and the Powellomyces proteins is shown in Figure 3. The *Olpidium* protein is a “fungal-type” TPPP as well since it contains the last part of the p25alpha domain in duplicate.

An abundant source of fungal sequences is the Mycocosm webpage [19] (https://mycocosm.jgi.doe.gov/mycocosm/home, accessed on 12 November 2021), which contains a great amount of additional data in comparison with the NCBI page. Thus, fungal-type TPPPs from this site were also compared with the *Olpidium* one (Figure 4). These proteins show a significant homology in their p25alpha domains, which does not hold in the interdomain part the length of which is also different by species. The Rossmann-like sequence is absent in some cases in the first (*Catenaria, Blastocladiella, Globomyces, Synchytrium*) or the second p25alpha domain (Batrachochytrium) but not in *O. bornovanus* (Figure 4).

**Table 1:** Accession Numbers of fungal proteins shown in Figure 4 and Figure 5.

Species	Accession No. ^1^
*Allomyces macrogynus*	KNE68590
*Batrachochytrium dendrobatidis* JAM81	XP_006680205
*Batrachochytrium salamandrivorans*	KAH6573313
*Blastocladiella britannica*	Blabri126943
*Blyttiomyces helicus*	RKO89614
*Caulochytrium protostelioides*	RKP02545
*Catenaria anguillulae* 1	ORZ33943
*Catenaria anguillulae* 2	ORZ35986
*Chytriomyces confervae* 1	TPX65513
*Chytriomyces confervae* 2	TPX72533
*Chytriomyces lagenaria* 1	Chylag1383254
*Chytriomyces lagenaria* 2	Chylag1491303
*Cladochytrium polystomum* 1	Clapol11821589
*Cladochytrium polystomum* 2	Clapol11869731
*Cladochytrium replicatum*	Clarep11774182
*Entophlyctis helioformis*	Enthel1467718
*Fimicolochytrium jonesii*	Fimjon1566472
*Gaertneriomyces semiglobifer*	Gaesem1531638
*Geranomyces variabilis*	Gervar1417039
*Globomyces pollinis-pini*	Glopol1609812
*Gorgonomyces haynaldii*	Gorhay1188404
*Obelidium mucronatum* 1	Obemuc1832726
*Obelidium mucronatum* 2	Obemuc1859513
*Olpidium bornovanus*	KAG5460860 + KAG5458366
*Paraphysoderma sedebokerense*	Parsed11082034
*Powellomyces hirtus*	TPX57673
*Rhizoclosmatium globosum* 1	Rhihy1315321
*Rhizoclosmatium globosum* 2	ORY45507
*Spizellomyces punctatus*	XP_016604112
*Synchytrium endobioticum*	TPX44587
*Synchytrium microbalum*	XP_031024160
*Triparticalcar arcticum*	Triarc1169044

^1^ Uppercase and lowercase accession numbers stand for NCBI and Mycocosm identifiers, respectively.

**Figure 5:**
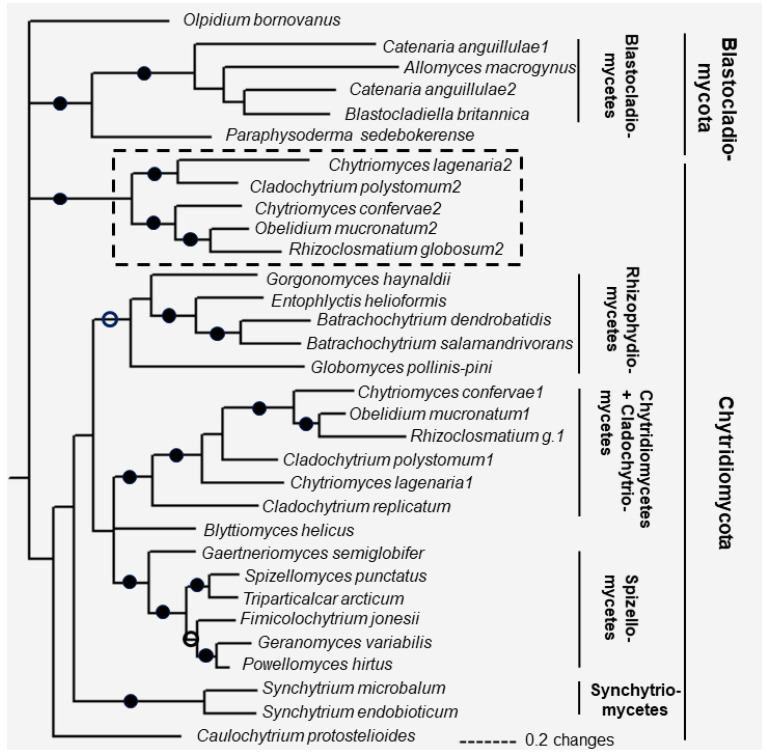
Phylogenetic tree of fungal-type TPPPs constructed by Bayesian analysis. Number of generations was 2.4 × 10^−6^. Full and open circles at a node indicate that the branch was supported by maximal Bayesian posterior probability (BPP) and ≥0.95 BPP, respectively. All the other branches were supported by BPP ≥ 50%. The box with dotted lines includes fungal-type TPPP paralogs being present only in Chytridiomycetes and Cladochytriomycetes. The Accession Numbers of fungal proteins are listed in Table 1.

Based on the multiple alignment of fungal-type TPPPs, phylogenetic analysis was carried out and a phylogenetic tree of fungal-type TPPPs was constructed using Bayesian method (Figure 5). *Olpidium*, not surprisingly, is separated from all other TPPPs which form three clades corresponding to the known phylogeny. One is the TPPPs of the phylum Blastocladiomycota, the second is those of the phylum Chytridiomycota, the third clade corresponds to a special group of paralogous TPPPs which are present only in certain families of Chytridiomycota (Chytridiomycetes and Cladochytriomycetes) [16]. Within the clades, the species phylogeny is valid, the sub-clades correspond to the various families: Blastocladiomycetes and Physodermatomycetes within Blastocladiomycota; Rhizophydiomycetes, Cladochytriomycetes, Synchytriomycetes and Spizellomycetes within Chytridiomycota. Here, TPPPs of Chytridiomycetes and Cladochytriomycetes form a common sub-clade as found earlier [16].

**Table 2:** Best protein hits when using *Olpidium bornovanus* KAG5460860 + KAG5458366 as a query.

Species	Max Score	Total Score	Query Cover	E Value ^1^	Percent Identy	Length	Accession
*Olpidium bornovanus*	322	322	61%	5 × 10^−109^	100.00%	163	KAG5458366
*Powellomyces hirtus*	186	186	100%	1 × 10^−53^	42.60%	303	TPX57673
*Spizellomyces punctatus*	179	179	100%	9 × 10^−51^	41.07%	315	XP_016604112
*Spizellomyces* sp. ‘*palustris*’	172	172	100%	6 ×10^−48^	38.54%	336	TPX61118
*Caulochytrium protostelioides*	171	171	100%	8 × 10^−48^	37.50%	319	RKP02545
*Chytriomyces confervae*	160	160	100%	2 × 10^−43^	35.88%	335	TPX65513
*Chytriomyces confervae*	155	155	100%	2 × 10^−41^	36.30%	335	TPX65886
*Synchytrium endobioticum*	150	150	99%	1 × 10^−39^	38.01%	338	TPX44587
*Olpidium bornovanus*	139	139	24%	2 × 10^−38^	100.00%	64	KAG5460860
*Synchytrium microbalum*	146	146	99%	2 × 10^−38^	36.26%	286	XP_031024160
*Batrachochytrium salamandrivorans*	124	124	85%	5 × 10^−30^	36.22%	286	KAH6573313
*Batrachochytrium dendrobatidis* JAM81	120	168	92%	2 × 10^−28^	36.23%	289	XP_006680205
*Batrachochytrium dendrobatidis* JEL423	120	168	92%	3 × 10^−28^	36.23%	299	OAJ42613
*Rhizoclosmatium globosum*	119	119	99%	3 × 10^−28^	36.23%	262	ORY45507
*Chytriomyces confervae*	117	117	90%	9 × 10^−28^	35.00%	255	TPX72533
*Chytriomyces confervae*	111	160	72%	4 × 10^−26^	40.44%	183	TPX78276

^1^ E-value is the measure of likeliness that sequence similarity is not by random chance. An E-value smaller than 1 × 10^−50^ includes database matches of very high quality. Blast hits with E-value smaller than 0.01 can still be considered as good hit for homology matches.

## 3. Conclusions

All the phyla of the flagellated fungi contain species with TPPP-like proteins except Aphelidiomycota; however, it can be expected that, similar to the case of Olpidiomycota, this deficit will disappear with a progress in sequencing of the members of the phylum. The occurrence of these proteins in Fungi varies according to phyla; the most early branching ones, Rozellomycota, Neocallimastigomycota, and Monoblepharomycota, do not contain the fungal-type TPPPs but other kinds of proteins of this family, such as apicortin, short- and long-type TPPPs [16]. In Chytridiomycota, which is the most well-known phylum of flagellated Fungi, all these proteins can be found, beside the most common fungal-type TPPP. Blastocladiomycota and Olpidiomycota seem to include species that possess only the fungal-type TPPP, featured by the presence of two (a complete and an incomplete) p25alpha domains. It is an open question whether this special TPPP was lost in the phyla closer to the root of the fungal phylogenetic tree, or whether they appeared only in more developed phyla. TPPP-like proteins, in general, are known to stabilize microtubules [13,21]. This protein is indispensable for the proper functioning of the flagellum, a microtubule-based organelle, in *C. reinhardtii* [14]. It has been shown that the amino acid sequences responsible for binding to microtubule are located at the C-terminus of the p25alpha domain [22,23]. Thus, the fungal-type TPPP contains two microtubule binding sites (cf. Figure 1), which may result in a stronger interaction. Whether it does cause a functional advantage requires further investigation. Through experimental work it can be verified whether TPPP is localized in the flagellum and the microtubule-TPPP interaction occurs in fungi, including *Olpidium*. However, the occurrence of a p25alpha domain-containing protein in *O. bornovanus* further strengthens the correlation suggested earlier [9,16] between the incidence of this domain and that of the eukaryotic flagellum.

## 4. Methods

Database homology search. It was carried out with an NCBI Blast search [24] (http://www.ncbi.nlm.nih.gov/BLAST/, accessed on 12 November 2021): sequences of various fungal proteins (e.g., *B. dendrobatidis* XP_006680205, *C. confervae* TPX65513, *P. hirtus* TPX57673, *S. punctatus* XP_016604112) containing p25alpha-domain were used as queries against protein and nucleotide databases to find similar sequences in *Olpidium* using BLASTP and TBLASTN analyses, respectively.

Phylogenetic analysis. Multiple alignments of sequences were done by the Clustal Omega program [20]. Bayesian analysis using MrBayes v3.1.2 [25] was also performed to construct phylogenetic trees. Default priors and the WAG model [26] were used assuming equal rates across sites. Two independent analyses were run with three heated and one cold chain (temperature parameter 0.2) for generations as indicated in the Figure legends, with a sampling frequency of 0.01 and the first 25% of generations were discarded as burn-in. The two runs were convergent. The phylogenetic trees were drawn using the program Drawgram of the PHYLIP package version 3.696 [27].

## Data Availability

All data are available in the paper and in the Supplementary Materials.

## Supplementary Materials

The following supporting information can be downloaded at: https://www.mdpi.com/article/10.3390/ijms232213927/s1. Data S1: Sequence alignment used for phylogenetic tree construction in nexus file and phylogenetic tree in con file.
