# Supplementary material for: On the TPPP Protein of the Enigmatic Fungus, Olpidium—Correlation between the Incidence of p25alpha Domain and That of the Eukaryotic Flagellum
Source: Int J Mol Sci. 2022 Nov 11;23(22):13927. doi: 10.3390/ijms232213927 (PMC9698843; doi:10.3390/ijms232213927)
Supplement: Supplementary file 1 [file ijms-23-13927-s001.zip › ijms-2000176-supplementary.pdf]

Data S1 Sequence alignment used for phylogenetic tree construction in nexus file.

```
#NEXUS
[TITLE: Written by EMBOSS 03/11/21]

begin data;
dimensions ntax=32 nchar=498;
format interleave datatype=protein missing=X gap=-;

matrix
Olpidium             XXXXXXXXXXXXXXXXXXXXXXXXXXXXXXXXXXXXXXXXKFAKFARETK
Catenaria1           lyqifeyfamfgv---sriaasg--------sdtvlidsarlaklfrdln
Allomyces            ----------------------------------------------mapv
Catenaria2           lrelfeqfasfgt---pranasgpn-----fqqetvmdsarflkffrdqn
Blabri126943         LHELFESFASFGA---SSSRPSVAA-----ATDGPLIDSARFVKFFRDTG
Chylag1491303        LEEAFIAFAAFG---SARKPRHGR------------SSGSRISKLARESG
Clapol1_1869731      LRSAFEAFASFG---SSRNLAASAP-IP--NAAATTIDSARFAKLARDTG
Chytriomyces2        saaafeefssfg---asrhpigspgpvph-haqgiamdsskfaklcrdsg
Obemuc1859513        LRTAFEEFALFG---SPRTQAHS---T---TTAPVQMDNAKFAKLCRDSG
Rhizoclosmatium2     lraafeefalfg---tpktiaprpahl---pnppiqmdskcfaklcrdsg
Gorhay1188404        LYESFDAFCQFG---SSRNLA---GSSTN-L--APQMDGAKFAKFCRDNK
Enthel1467718        LYNCFSSFCQFG---SSRNLSS-MGSMTDLA--GPTMDGARWAKFCRDSG
Batrachochytriumd3   lymtfgsfcqfg---ssrnls---gsmtdis--gptmdgskwakfcrdtg
Batrachochytriums1   lytifasfcqyg---ssrnls---gsasdis--gptmdgskwakfcrdsg
Parsed11082034       LYEVFTQFCAFGAS--RNSNPNLSGSLNNLS-SGPQIDNVRFSKFFRDLK
Glopol1609812        LYGVYESFCQFGST--RTSNASLSSSLNNLK--GPTMDGSKWAKFCRDCK
Synchytriumm         lrtvydsfcafg---ssrnlssnsmsmeslt--gaqldnakfaklskdtg
Synchytriume         lrdryeafcafg---ssrnlagaatsi-dig--ghqmdnakfakftkdtg
Chytriomyces1        lharfekfcafg---rgsvgssldsl-----tagstmdgakwakfardcn
Obemuc1832726        LRNTFERFCAFG---RGSVGSSLDSL-----TGGSTMDGAKWAKFARDSN
Rhizoclosmatium1     LRSTFEKFCAFG---RGSVGSSLDSL-----HGGSTMDGPKWAKFARDSN
Chylag1383254        LHDVFERFAAFG---SNRNLSSLDSSLLSISGGGLTLDGPRFAKFARDTG
Clapol1_1821589      LHDVFERFCSFG---SNRNLASMDASF----GPGLTMDGAKFAKFARDSG
Blyttiomyces         XXXXXXXXXXXXXXXXXXXXXXXXXXXXXXXXXXXXXXXXXXXXXXXXXX
Caulochytrium        lrvvfesyanfg---asrnvas-ngslnnla--gpqldnakfaklcretk
Clarep1_1774182      LRSVYETFCNASGSSSRMNLASPHSSLSDLSGAGLTMDGARFAKFARDCK
Gaesem1531638        LREIYEAFCAFG---SNRNLSS-TGSLENIA--GPQMDGAKFAKFARDNK
Spizellomyces1       lreiyeafcafg---snrnlsnphgsmesia--gptmdgakfakfardnr
```

Triarc1169044	LREIYDAFCAFG---SNRNLSNPHGSLESIA--GPTMDGAKFAKFARDNK
Fimjon1566472	LREIFDAFCAFG---SNRNLATG-----EAG--GNTMDGAKFAKFARDNK
Gervar1417039	LKEVYVAFCAFG---SNRNLASS----SDVQ--GPTMDGAKFAKFARDNK
Powellomyces	lreiyeafcafg---snrnlats----tdvs--gptmdgakfakfardnk

Olpidium	LISAPKVTAVDVDNAFMRVK---PRASRKIDFEGFQQALRILAEKRYP-Q
Catenaria1	lfdk-sfnsialdinfskakatfqrpdrklpwaafiyalqlsaankypkl
Allomyces	lrpp-tftttdldlifnkakvhfrrqdrklhydaflyalklsafakfggk
Catenaria2	lldt-nltptsldlhftkakasfkrtdrklpwnaflyalqlaattkygsa
Blabri126943	LLDS-HLTQTTLDIHFAKAKSQFKRADRKLPFDAFLYALQLAAVSKYGGA
Chylag1491303	MIGG-NLTVTDVDIVFGRCKV-----GRKLDFKAFKKALEMIAERRGC--
Clapol1_1869731	LIAPPSLTIVDVDLIFEKCKI--AKGTRKLDFKGFKKALQMVAEKRQC--
Chytriomyces2	lidnetftlidcdivfskckk---vgkrkleyddfrralaeiadirqc--
Obemuc1859513	LVDNISLSLIDVDIIFAKCKQ---VGKRKLFYADFQRALREIADTRQC--
Rhizoclosmatium2	liddislslidvdiifakvkq---pgkrklgyadfqralreiawtrqc--
Gorhay1188404	LIGG-KVTPTEVDIVFNKVK---GKTARKIDFKEFQEGLRLLAAKKYEG-
Enthel1467718	LLDK-DISATDIDIMFNKVK---AKTARRIDFDQFQAALRLIAAKRFGNS
Batrachochytriumd3	iidk-hitttdidiwfnkvk---aktvrkidfeqfqaalhlvaakrygss
Batrachochytriums1	figk-sitatdfdiwfnkvk---aktarkidfdqfqaalhlvaekrygan
Parsed11082034	VLNK-ELTPTDIDIIFNKVKK--TKTDRKLDFDCFKNALVLCSEKKYRPK
Glopol1609812	VIDK-VITPTEVDIVFNKVK---SKTERKIDYDQFIEGLKMISAKKYS-N
Synchytriumm	vvdgkkitnadiditfnkvk---akgarkldwesfqdalallaekkya-t
Synchytriume	ivdk-rvtiidvdiifnkvk---akgarkldwetfqdalahlaekkyp-s
Chytriomyces1	lidnkrvtstdidiifnkvk---akstrridweefqvavklvagkkyp-k
Obemuc1832726	LIDNKKITSTEIDIIFNKVK---AKNSRRIDWEEFQAALKMVAEKKYA-E
Rhizoclosmatium1	LIDNKKITSTDIDIIFNKVK---SKNSRRIDWDEFLCAVKMVAEKKYV-G
Chylag1383254	LVDGKKVTATDIDIIFNKTK---SKGNRRIDWPVFLEAVQLIAEKRYP-E
Clapol1_1821589	LIDGRKVTTTEIDIIFNKVK---AKTARRIDWNEFVKAVQMLAEKRYP-N
Blyttiomyces	XXXXXXXXXXXXXXXXXXXXXXXXXXXXXXXXXXXXXXXXXXXXXXXXXX
Caulochytrium	vvdgkrittteidisfskvl----kggrkmdfgqfqevlrllaekkfg-g
Clarep1_1774182	LIDGKRVTTTDVDIIFNKAK---AKGSRRLDWTTFQAAVRALAQKKYP-E
Gaesem1531638	IIDGKKVTTTDVDITFNKVK---PKGARKLDWNTFLDALTQLAEKKYA-G
Spizellomyces1	lidnkrvtttdvdiifnkvk---tkgarkldwntfidglteiavkkyp-g
Triarc1169044	LIDNKRVTSTDVDITFNKVK---PKGSRKLDWNTFLDGLTDIAEKKNP-G
Fimjon1566472	LIDNKKVTSTDVDIVFNKVK---PKGARKIDWNGFIEGLTLVAEK-KT-G
Gervar1417039	LIDNKKVTSTDVDIIFNKVK---PKGARKLDWNTFLEGLTQIAEKKNP-G
Powellomyces	lidnkkvtstdvdiifnkvk---pkgarkldwntflegltqiaekkns-g

```
Olpidium              KDPE-----------EAY----LTLVNHVCQSKGPKVHSI---Q------
Catenaria1            see------------eafsklvheiirhpgpdfapdypgga---------
Allomyces             pasdpadplaaiaeqailenlvseicatg--ilapvvrsaa---------
Catenaria2            msda-----------daleqlineavksq----gpiaratv---------
Blabri126943          MSEN-----------EALEQIIIEACNGS----GPIARGTV---------
Chylag1491303         ----------------TIEHVKALVI-GS----EPQLKGTV---------
Clapol1_1869731       ----------------DYSHVVAIVV-AG----SPALTGTI---------
Chytriomyces2         ----------------hvshvvamvv-sa----apeingtl---------
Obemuc1859513         ----------------DIDHVVAMVV-TA----APELNGTI---------
Rhizoclosmatium2      ----------------deehvvalvv-sa----pvlingti---------
Gorhay1188404         KNPI-----------EQFNAIVRHITSGDN---KPIAYATL---------
Enthel1467718         KSPT-----------EAFAAIIRTIVNGNV---RPVLQGTVRRSAGCGTA
Batrachochytriumd3    kppt-----------eaynllvrsilnsga---rpvatgti---------
Batrachochytriums1    kspt-----------eaymliirditnggt---rpvtiats---------
Parsed11082034        MDGY-----------QAYEKIVTDIVSKDN--AGPLLHGT---R------
Glopol1609812         KTTT-----------ESFVILVQFVLSTRG---APVVQTNLKNS------
Synchytriumm          lpae-----------qafsrlvtdilsngs----phltnt--av------
Synchytriume          lqpe-----------qgfnklvsdvlassg----psltgt--av------
Chytriomyces1         mheq-----------daytktiydacilsn---gpvarat---t------
Obemuc1832726         KHAQ-----------DAFTQVMYDVCVRAH---GPVSKAT---A------
Rhizoclosmatium1      MHEQ-----------DAFTQVMYDVCVRSG---GPAVKAT---A------
Chylag1383254         KSDQ-----------QALHCTLYDVCIRSP--GGPAKKGT---T------
Clapol1_1821589       KRPQ-----------DALDSTLYDVCIKAK---GPVSSGT---A------
Blyttiomyces          kkgn-----------qafdalikdlsrksv---gpvssgt---v------
Caulochytrium         avd-------------aqqkliaqic-qsk---gpkasgt---t------
Clarep1_1774182       RAEM-----------EAFNTVMLEVTQARG---PLINKAT---T------
Gaesem1531638         KKGR-----------EALEAVTGDIVKRGS---GPIASGTVRDT------
Spizellomyces1        kqgr-----------qaldavistimkkgg---gpiatgt---t------
Triarc1169044         KKGR-----------DALDIVIGTIMKKGG---GPIATGT---T------
Fimjon1566472         KQGR-----------DALDAVIGLILKKGG---GPIATGT---A------
Gervar1417039         KQGR-----------DALDSLIGLIIKKGG---GPIATGT---A------
Powellomyces          kqgr-----------ealdsligliikkgg---gpiatgt---a------

Olpidium              ---------------------------------PEATGIFEKLTDTKL--
Catenaria1            ---------------------------------raqdgaaapvsrasrrs
Allomyces             ---------------------------------adsvapfsaaatap---
```

```
Catenaria2             ---------------------------------pvadgvygkltdasq--
Blabri126943           ---------------------------------TATGGIYSKLTDPTL--
Chylag1491303          ---------------------------------PDMIPIVEKLTDASL--
Clapol1_1869731        ---------------------------------PDYDPVIEKLTDTSL--
Chytriomyces2          ---------------------------------phadelidkltdtsl--
Obemuc1859513          ---------------------------------PQVTELIEKLTDTSL--
Rhizoclosmatium2       ---------------------------------pqvdeitekltdvsl--
Gorhay1188404          ---------------------------------PANDSITQRLTDTTA--
Enthel1467718          RVLTDGCLIVAGSWLLADRGCWLVARGCWMAQAASNDAVTQRMTDHTL--
Batrachochytriumd3     ---------------------------------ttsdsvtqrltdhth--
Batrachochytriums1     ---------------------------------ttndavtqrltdhtq--
Parsed11082034         ---------------------------------PEASGVYSKLTDHTL--
Glopol1609812          ---------------------------------VQ-MDVTNRLTDPQK--
Synchytriumm           ---------------------------------pknsaivdrltdtst--
Synchytriume           ---------------------------------sknaaivdrltdast--
Chytriomyces1          ---------------------------------vkndavldrltdvsg--
Obemuc1832726          ---------------------------------VQKDAVLDRLTDVNG--
Rhizoclosmatium1       ---------------------------------VKNDAVLDRLTDVNG--
Chylag1383254          ---------------------------------VQADAVLDRLTDTTA--
Clapol1_1821589        ---------------------------------VKNDAVLDRLTDTHA--
Blyttiomyces           ---------------------------------aqtdavldrltdtqa--
Caulochytrium          ---------------------------------pdakgahnrltdtsq--
Clarep1_1774182        ---------------------------------AKTDGVYDKLTNTQL--
Gaesem1531638          ---------------------------------PQSDAIVDRLTDTSK--
Spizellomyces1         ---------------------------------pksdgivdrltdtsk--
Triarc1169044          ---------------------------------PKNDAITDRLTDTSK--
Fimjon1566472          ---------------------------------PKSDAIVDRLTDTSK--
Gervar1417039          ---------------------------------PKSDAIVDRLTDTSK--
Powellomyces           ---------------------------------pksdaivdrltdtsk--

Olpidium               Ytgahkarfdd-ngvgrgaagqvdaedatrnlstivsrdpaskqk-ravs
Catenaria1             stmprrtsaes-ndsgigesages-sg--------------sfgsgsg--
Allomyces              cdaprr-------sksthqlhdss-tkiasss--------------sk--
Catenaria2             ytgqhk-------dkpsgqsegkg-ytiesplaksr-a------tgsr--
Blabri126943           YTGQHK-------DKPTGSSGGAS-REVTIEESRTS-RSKSRSRPGSR--
Chylag1491303          YTGSHRERFDSVTGVGRGAAGRDT-VAKTGSLSDIVSRHNPNTTLGSPKV
Clapol1_1869731        YTGTHKNRFDPETGVGRGLEGREP-VNTTSNLANLVSRHNPNKTLGAPTP
```

```
Chytriomyces2        ytgmhrarfdpvtgqqrglagret-isptadlsaivsrhnsattlgskas
Obemuc1859513        YTGTHKARFDPVTGQGLGLDGREP-VNPTANLSSIVSRHNTARTLGSKAL
Rhizoclosmatium2     ytgthkerfdpvtgqqrglagrdt-inttanlstivsrhntartlgskgp
Gorhay1188404        YTGTHKNRFDE-NGRGLGMQGRDP-K--TISLSNLANREPADIRG-VNFS
Enthel1467718        YTGTHKNRFDA-SGNGLGMSGRDQ-PSRTNDLAKITNREESNVRG-VPLS
Batrachochytriumd3   ytgthknrfde-agqglglagrdt-hsrtnelskivnrkeadirg-vpla
Batrachochytriums1   ytgthkgrfgs-ngnglgmagrdt-nsktnelskivnrkeanvrg-vsid
Parsed11082034       YTGSHKERFDE-EGRGKGVSGRNDSSIGVKRLDKLVSRDPAVQNR-AASM
Glopol1609812        YTGIHKEKSHG-T----MGSVYDLSDSGRESISTIKTTNG----------
Synchytriumm         fpvthknrfed-kglgsqgalna---------ksstg---------ttpt
Synchytriume         fpvthknrfee-hgvlksspfgas----reglgksng---------ttpt
Chytriomyces1        ytgthknrfds-agrglglngrvt-gkstdtlskivnrdapsqqp-laqt
Obemuc1832726        YTGSHKNRFDS-AGRGLGLQGRDT-ISRTDTLGKIVSRDIAVQRP-SVPQ
Rhizoclosmatium1     YTGSSKNRFDS-AGRGLGLQGRDT-INRTDTLSKIVNRDAPLQRS-TTVG
Chylag1383254        YTGTHKSRFDE-NGNGRGLAGRDT-GSKTDRLDKLVSRNDSTT-------
Clapol1_1821589      YTGTHKLRFDA-DGHGRGMEGRDQ-PSKTDRLDKLVNRDGAGT-------
Blyttiomyces         ytgthklrfdd-eghglgmagrdq-psktndlakianreatslrg-lpvs
Caulochytrium        ytgthklrfde-egrgrglagrda-paptadlsqildrsgatvrg-vplk
Clarep1_1774182      YTGAHKLRFDE-QGNGRGMEGLKE-RE-----------------------
Gaesem1531638        YTGTHKLRFDE-QGHGRGLAGRDQ-PAKTDDLSKLVNREPTTVRG-LPVS
Spizellomyces1       ytgthklrfde-agrgrgaegrdr-psatdqlskitnreetsvrg-lpvs
Triarc1169044        YTGTHKLRFDE-AGRGRGAEGRDR-ASATDQLSKITNREETSVRG-LPMS
Fimjon1566472        YTGTHKLRFDD-AGHGRGAAGRDR-PTATADLSQITNREETSVRG-LPVS
Gervar1417039        YTGTHKLRFDE-AGQGRGAAGRDR-PSPTSDLSKITNREETSLRG-LPVS
Powellomyces         ytgthklrfde-agqgrgaagrdr-psatsdlskitnreetslrg-lpvs

Olpidium             n-------------------------------------------------
Catenaria1           ------------------------slsrsrsltrsrs-----------ss
Allomyces            ----------------------------sasran---------------l
Catenaria2           ----------------------------snstsnlld-----------as
Blabri126943         ------------------------SASRSGSVSDLTS-----------AL
Chylag1491303        M-------------------------------------------------
Clapol1_1869731      P-------------------------------------------------
Chytriomyces2        a-------------------------------------------------
Obemuc1859513        P-------------------------------------------------
Rhizoclosmatium2     l-------------------------------------------------
Gorhay1188404        LKPSSSNSSLSNHGSKSNLTTGSKSNLTTGSKSNLTTGSKSNLATKIVQQ
```

Enthel1467718	AAYGSNSNMADGRYSTSSITPSIHNS----S--------N-SSHVGNGTS
Batrachochytriumd3	aspigsprg----sv-----------------------------tmsysp
Batrachochytriums1	atdssqskg----yt-----------------------------a---at
Parsed11082034	NQTN--
Glopol1609812	--RRYSN--------------------------------------FGQET
Synchytriumm	kspggg---v
Synchytriume	kspat--v
Chytriomyces1	nrqsvsnrrqesqst---------------------------mraptaas
Obemuc1832726	NSRPIT------------------------------------TTEKNRTS
Rhizoclosmatium1	GTRRPTTVA---------------------------------QADSKQGT
Chylag1383254	--
Clapol1_1821589	--
Blyttiomyces	adpaaga-----------------------------------ssasrnat
Caulochytrium	taertan-----------------------------------ttata--a
Clarep1_1774182	--RSN-AG
Gaesem1531638	VDPDAQE-----------------------------------QS-KP-SS
Spizellomyces1	idpeegd-----------------------------------kqelt-ss
Triarc1169044	IDPQEKD-----------------------------------KT------
Fimjon1566472	IDPQERG-----------------------------------AS------
Gervar1417039	IDPNEKS-----------------------------------SP------
Powellomyces	idpnekg-----------------------------------sp------

Olpidium	---------------saealdnkppvk--------kysavrs--snksln
Catenaria1	sgtsfsaara------------------------------------psvp
Allomyces	ggaggs--------------------------------astrgsqs-nlm
Catenaria2	tgtrg------------------stgaln------kstssprgsk-snly
Blabri126943	AGASISTPRAAAATASFSTLSSTSAGPSA------TTGHSPRASRTSLAY
Chylag1491303	---------------RVATITPPKSPP--------TT-------------
Clapol1_1869731	---------------KSPAMSRTASTP--------NRSGS----------
Chytriomyces2	---------------anralspp---------------------------
Obemuc1859513	---------------TPHASHPSSPT------------------------
Rhizoclosmatium2	---------------spsssfsasapi--------ia-------------
Gorhay1188404	PKKREHDN---IVTASSETLDKQMAKP--------KR-------------
Enthel1467718	SGKRGHQS---VVTASSERLDLQSSKP--------KKVGSPVKRSTSNLA
Batrachochytriumd3	kskrahaa---vttmstealdaeanrp--------kkavtsqapr--tvk
Batrachochytriums1	ipkrthqs---vstvssealdakaskp--------kkglppiagr--ses
Parsed11082034	-------K--RGISASMEAMDAQGPTKSIKNLELPNDASLSH--SRSNSG

```
Glopol1609812         STKRGF-N--NVVTASTEYLDMNSNKT--------KKQEMYS--SNTQLP
Synchytriumm          kgspvgnk--rsvtesmdklelaanpp--------ksnkalg--smdnms
Synchytriume          klgvsgnk--rgvtestekldmsknpv--------ksnknmg--smd-ma
Chytriomyces1         sisqnkra--svltqseeklenivnap--------kkapvag--rrsqap
Obemuc1832726         TVASGKRQ--SVLTQSEERLENITNAP--------KKTPVAG--RRATQ-
Rhizoclosmatium1      VVGSSKRP--SMLTNSEERLENQINAP--------KKTPIAG--RRATT-
Chylag1383254         -PTIP---------PISARLEIIATTP--------KK-P-----------
Clapol1_1821589       -AASTKRQ--QVLTQSEEQLDAIAQAP--------KK-AIAG--RRAANP
Blyttiomyces          pgkrshas---vvtassekldltaskp--------kkadarr--sntnla
Caulochytrium         gtrrtatq--rtplgsqgslsastppp--------krvtptp--ststrp
Clarep1_1774182       GQKRGQTH---IVTASSERLDLVAHAP--------KRSADSA--SK----
Gaesem1531638         GSKRGHAS---VVTASSEKLDVNAHKP--------KATKPAE--KTKKA-
Spizellomyces1        agkrghss---vvtasserldlasskp--------kssklgs--ntnla-
Triarc1169044         -SKRSHSN---LATASSDRLDQSSSKP--------KSTKKGS--NTNLA-
Fimjon1566472         -AKRSHSN---LVTASSERLDREASKP--------KNTKVGS--NTNLA-
Gervar1417039         -AKRGHSS---VVTASSERLDQAASKP--------KAGRIGG--SNSNL-
Powellomyces          -akrqhsn---vvtasserldnaasqp--------kssklgs--nsnla-

Olpidium              r----aggsrdrpas----------------------------kseaap-
Catenaria1            vpp---------------iprsksptrarmslfgaq--------------
Allomyces             aat---------------aasaahattstrsrpasr--------------
Catenaria2            apt---------------tssna-----kiagskgs--------------
Blabri126943          KPT---------------ASSTS-----RMSLAASG--------------
Chylag1491303         --------------------------------------------------
Clapol1_1869731       --------------------------------------------------
Chytriomyces2         --------------------------------------------------
Obemuc1859513         --------------------------------------------------
Rhizoclosmatium2      --------------------------------------------------
Gorhay1188404         --------------------------------------------------
Enthel1467718         N------IKPSEPTP-----------------------------------
Batrachochytriumd3    p------ivkssetl-----------------------------------
Batrachochytriums1    n------nklsdptp-----------------------------------
Parsed11082034        A--SSNGLNKSNSNL----------TRSKSSGL-----------------
Glopol1609812         KVNFG--SK-----------------------------------------
Synchytriumm          p-------------------------------------------------
Synchytriume          s-------------------------------------------------
Chytriomyces1         htttkggnytasssl------------------assq-------------
```

```
Obemuc1832726        ----QSANYTTSSSL------------------ASSQ-------------
Rhizoclosmatium1     ----HDSSSGNYSTI------------------QPKL-------------
Chylag1383254        TNRSIHGSSTSI--------------------------------------
Clapol1_1821589      VPSSKFGSSTSIKS--------------------STP-------------
Blyttiomyces         akaattparrpnpslagpaassssttrrsnqdlsstpastrasradrapa
Caulochytrium        aaaktaaaskaaatk-----------------------------------
Clarep1_1774182      --P-----------------------------------------------
Gaesem1531638        EPK---------P-Y-----------------------------------
Spizellomyces1       nkpktpqak-----------------------------------------
Triarc1169044        SKSSPA--------------------------------------------
Fimjon1566472        GKPKTAGG----GNA-----------------------------------
Gervar1417039        NKGSSSNLSKQSSSV-----------------------------------
Powellomyces         g--------kpkpps-----------------------------------

Olpidium             ---------------------ksagaprsaaasadknvfdrltdpkgyrg
Catenaria1           ---psgdaqatpatgarkhqlgsadfvptvpviprgsvfdrltnprgytg
Allomyces            ---tgsrt-nlaagagskhelgsveftptvpsipkgsvfdrltnpskytg
Catenaria2           ---lagrt-kgaagtgshttlgsseyvptqpsipkgsvydrltnpkgyta
Blabri126943         ---ATGGS-R-GNLTGSNAKLGSPDYVPTVPSIPKGSVFDRLTNTKGYTG
Chylag1491303        ------------------------PRMQSPRTTNGQSVFDRLSSPTTFTG
Clapol1_1869731      ---------------------EGSLGSAPSSPSARPSVFDRLTSVSTFTG
Chytriomyces2        -----------------------phertaasssagpsvfdrlnnvksftg
Obemuc1859513        -----------------------SPVRYSEDQGGRESVFDRLNNVSTFTG
Rhizoclosmatium2     ---------------------wgenrsmsgsedgkpsvfdrlndvstftg
Gorhay1188404        --------------------------------PEGNSVFDRLTDTAKYTG
Enthel1467718        ---------------------LSSLNKRSTSGSMGSSVFERLTDTKAYTG
Batrachochytriumd3   ---------------------lntttkktvgas-sgnvfdrltntkgytg
Batrachochytriums1   ---------------------ltasakkaavgs-sgnvydrltdtkgytg
Parsed11082034       -------------SG------SSSQLKTTVPQVPAGSVFDRLTDVKKYHG
Glopol1609812        ---------------------Q-NLTSSNSNLNQNGNVFDRLTNTQGYTG
Synchytriumm         -----------------------l-gasksksgsvgsvydrltdtkqytg
Synchytriume         -----------------------l-sasksksgsvgsvydrltdhksytg
Chytriomyces1        ---------------------qslaks--sttasklsvfdrltdssgytg
Obemuc1832726        ---------------------QSLATKNSSVSSSKASVFDRLTNTKDYTG
Rhizoclosmatium1     -----------------------LGTT--------QKLIHDIVQGKRI--
Chylag1383254        ---------------------------ASVGKSTGSSVFDRLTNSNGYTG
Clapol1_1821589      ---------------------GSITTLNKSVTGSRTAVFDRLTDTSGYTG
```

Blyttiomyces	aasttrrsntdlasgarrtvatpaskgtynasgtnssvfdrltttagytg
Caulochytrium	---------------------tagkay-aasspaggsvfdrltntagytg
Clarep1_1774182	------------------------TTAKTGAQKGGTNVYDRLTNTGAYTG
Gaesem1531638	---------------------TAVTKTSCGQSSKGGSVFDRLTDSSGYTG
Spizellomyces1	----------------------idksyg--anakggsvfdrltnsgqytg
Triarc1169044	-----------------------AKSEE--YTAKGGSVFDRLTDSNQYTG
Fimjon1566472	---------------------AAASSYGAS-SSKGGSVFDRLTDSSGYTG
Gervar1417039	---------------------EAKQSYGTANAKAGGSVFDRLTDTTGYTG
Powellomyces	---------------------asaqsygtnnpkaggsvfdrltdssgytg

Olpidium	thaqrfde-qgkgrglagrdstskga-gpgayhggdvkdisqilrs--
Catenaria1	thkerfda-dgrgkgkagrvetesvq-----------mtlsemvkrf-
Allomyces	thkerfdpatgkgrglagrsqenlgt-----------kpldklvhrs-
Catenaria2	thkerfdd-qgkgkgkagrvqddlgt-----------kslehlvark-
Blabri126943	SHRERFDE-DGRGRGKAGRVQENVGT-----------QSLEKLVSRN-
Chylag1491303	THKHRFNP-DGTGRGLEGRTGEGAGE---------TVNSLSQITRK--
Clapol1_1869731	SHKHRFNA-DGTGRGKEGRVGDGAGD---------VVSDLSQITRR--
Chytriomyces2	thkhrfns-dgtgrgkagrd----------------------------
Obemuc1859513	THKHRFNE-DGTGRGKAGRED---------------------------
Rhizoclosmatium2	thkhrfnp-dgtgrgkegrdspssta---------d---lsqivsark
Gorhay1188404	THKERFNA-DGSGRGIAGRDTVGQPY----LYRT---GSSSSITRA--
Enthel1467718	THKHRFND-DGSGRGIDGRDPNAGTS----GTKT-----LASILRT--
Batrachochytriumd3	thkerfhg-ssn--sihg------------------------------
Batrachochytriums1	tqkerfgg-sks--sirg------------------------------
Parsed11082034	THKHRFNE-DGSGKGKAGRVQDVGP------------KKLESFLRN--
Glopol1609812	THKQRFDE-HGNGRGLEGREAIAKGSGTQSIYRGGNVNSLSQILRS--
Synchytriumm	thklrfnd-dgtgrgiagrdspskgg-vgpvrdgqnvndlsqilrr--
Synchytriume	thklrfnd-dgsgrgiagrdspskgg-tgvvrdgqnvndlsqilrr--
Chytriomyces1	thqhrfna-dgsgrglagrdsaplgkggvtqyrggnvnslsqilrs--
Obemuc1832726	AHKHRFNA-DGTGRGLAGRDSAPLGSGGESKYRGGDVKDLKQILRT--
Rhizoclosmatium1	--
Chylag1383254	THKLRFNA-SGTGRGLAGRDSIPK-N-GVGVYRGGDVKELSQILRPGF
Clapol1_1821589	SHKHRFNA-DGTGRGAAGREIVSKGH-SVGTYRGGDVKDLSQILRN--
Blyttiomyces	thklrfna-dgtgrgmagrdapakga-apgayrggdvkdlsqilrs--
Caulochytrium	shkerfna-dgtgrglagreapnksg-apgtyrggnvsdlsqilrt--
Clarep1_1774182	THKLRFDA-SGQGRGLAGRDSAPKGQ-SAGQYRGGDVKDLSQILRN--
Gaesem1531638	SHKERFNS-DGTGRGLSGRDSPSKSG-AAGKYRGGDVKDLSQILRS--

```
Spizellomyces1      thkhrfna-dgsgrgiagrdspakgt-spgsyrggdvkdlsqilrs--

Triarc1169044       AHKHRFNA-DGSGKGIVGRDSPSKGG-GPGTYRGGDVKDLSQILRS--

Fimjon1566472       AHKQRFNA-DGTGKGLAGRDSPAKGG-APGKYRGGDVKDLAQILRS--

Gervar1417039       AHKQRFNA-DGTGRGIAGRDAPAKGG-SPGKYRGGDVKDLSQILRN--

Powellomyces        ahkqrfna-dgtgkglagrdapakgn-spgkyrggdvkdlsqilrn--

;


end;

begin assumptions;

options deftype=unord;

end;
```

```
Phylogenetic tree in con file.

#NEXUS


[ID: 1928394251]

begin trees;

   [Note: This tree contains information on the topology,

          branch lengths (if present), and the probability

          of the partition indicated by the branch.]

   tree con_50_majrule =
(Olpidium:0.491849,((Catenaria1:0.636440,(Allomyces:0.616783,(Catenaria2:0.319621,B
labri126943:0.297141)0.71:0.075056)0.51:0.099658)1.00:0.365325,Parsed11082034:0.420
645)1.00:0.220331,((Chylag1491303:0.463140,Clapol1_1869731:0.209358)1.00:0.160893,(
Chytriomyces2:0.229313,(Obemuc1859513:0.133213,Rhizoclosmatium2:0.249109)1.00:0.121
602)1.00:0.153875)1.00:0.363354,(((((Gorhay1188404:0.370528,(Enthel1467718:0.231303
,(Batrachochytriumd3:0.244778,Batrachochytriums1:0.211146)1.00:0.155674)1.00:0.1571
77)0.54:0.072211,Glopol1609812:0.609138)0.98:0.136199,(((((Chytriomyces1:0.207280,(
Obemuc1832726:0.090743,Rhizoclosmatium1:0.303189)1.00:0.088831)1.00:0.252477,Clapol
1_1821589:0.190135)0.62:0.074434,Chylag1383254:0.286597)1.00:0.172461,Clarep1_17741
82:0.369318)1.00:0.138789,Blyttiomyces:0.287482,(Gaesem1531638:0.161047,((Spizellom
yces1:0.101310,Triarc1169044:0.100769)1.00:0.080855,(Fimjon1566472:0.130326,(Gervar
1417039:0.118654,Powellomyces:0.041193)1.00:0.067198)0.99:0.040848)1.00:0.129354)1.
00:0.127557)0.70:0.072532)0.54:0.066087,(Synchytriumm:0.183318,Synchytriume:0.18148
6)1.00:0.459595)0.73:0.070564,Caulochytrium:0.447358)0.77:0.088088);


   [Note: This tree contains information only on the topology

          and branch lengths (mean of the posterior probability density).]

   tree con_50_majrule =
(Olpidium:0.491849,((Catenaria1:0.636440,(Allomyces:0.616783,(Catenaria2:0.319621,B
labri126943:0.297141):0.075056):0.099658):0.365325,Parsed11082034:0.420645):0.22033
1,((Chylag1491303:0.463140,Clapol1_1869731:0.209358):0.160893,(Chytriomyces2:0.2293
13,(Obemuc1859513:0.133213,Rhizoclosmatium2:0.249109):0.121602):0.153875):0.363354,
(((((Gorhay1188404:0.370528,(Enthel1467718:0.231303,(Batrachochytriumd3:0.244778,Ba
trachochytriums1:0.211146):0.155674):0.157177):0.072211,Glopol1609812:0.609138):0.1
36199,(((((Chytriomyces1:0.207280,(Obemuc1832726:0.090743,Rhizoclosmatium1:0.303189
):0.088831):0.252477,Clapol1_1821589:0.190135):0.074434,Chylag1383254:0.286597):0.1
```

```
72461,Clarep1_1774182:0.369318):0.138789,Blyttiomyces:0.287482,(Gaesem1531638:0.161
047,((Spizellomyces1:0.101310,Triarc1169044:0.100769):0.080855,(Fimjon1566472:0.130
326,(Gervar1417039:0.118654,Powellomyces:0.041193):0.067198):0.040848):0.129354):0.
127557):0.072532):0.066087,(Synchytriumm:0.183318,Synchytriume:0.181486):0.459595):
0.070564,Caulochytrium:0.447358):0.088088);

end;
```
